# AI applications in veterinary digital health: a systematic survey

**DOI:** 10.3389/fvets.2026.1853395

**Published:** 2026-07-30

**Authors:** Sameera Polapragada

**Affiliations:** Independent Researcher, Lake St. Louis, MO, United States

**Keywords:** animal health, artificial intelligence, deep learning, machine learning, systematic survey, veterinary diagnostics, veterinary digital health, veterinary medicine

## Abstract

Veterinary medicine is experiencing a digital transformation driven by Artificial Intelligence (AI) applications in diagnostic imaging, disease prediction, clinical decision support, wearable monitoring, and telemedicine. This systematic survey, following the PRISMA 2020 reporting framework, synthesizes research published between 2013 and 2025 to categorize the taxonomy of AI applications, from convolutional neural networks for radiography to large language models (LLMs) for clinical consultation, and to evaluate the current state of AI and deep learning (DL) in veterinary digital health. Using predefined eligibility criteria, 22 studies met the inclusion criteria, comprising 18 primary evidence studies and 4 supporting domain/prototype studies. These studies were classified according to major application domains, including diagnostic imaging, predictive analytics, wearable monitoring, livestock health management, clinical decision support, and emerging LLM-based veterinary systems. The included studies indicate significant advancements in automated radiography, cytology, cardiovascular disease detection, dermatology, oncology, and clinical decision support, highlighting the potential of AI to enhance diagnostic accuracy, early disease detection, and clinical efficiency. Nevertheless, widespread clinical adoption is limited by fragmented datasets, species diversity, insufficient external validation, the opaque nature of many AI algorithms, and the absence of prospective real-world clinical evaluation. While LLM-based veterinary applications exhibit considerable promise, their implementation is still in its early stages, highlighting the necessity for rigorous validation prior to routine clinical use. This survey offers a structured synthesis of current AI applications in veterinary digital health and identifies key methodological limitations, implementation barriers, and future research priorities. Overall, the findings emphasize the importance of standardized datasets, robust model validation, explainable AI (XAI), and interdisciplinary collaboration to ensure the safe, reliable, and ethical integration of AI into veterinary medicine.

## 1 Introduction

Veterinary healthcare remains underserved compared to human healthcare due to fragmented data repositories, limited centralized clinical datasets, reduced access to specialized veterinary services in rural regions, the absence of comprehensive regulatory frameworks for Artificial Intelligence (AI)-enabled veterinary tools, and slower translation of research into clinical practice (1, 6). Concurrently, the increasing adoption of telemedicine and wearable technologies reflects growing demand for digital veterinary care and proactive health monitoring among pet owners (7, 8).

Recent advances in AI have facilitated veterinary applications in diagnostic imaging, automated triage, predictive epidemiology, disease surveillance, dermatological assessment, chronic disease prediction, and continuous welfare monitoring through wearable technologies (2, 4, 9, 10). These innovations suggest considerable potential to improve diagnostic accuracy and clinical decision-making. However, the integration of AI into routine veterinary practice remains limited as most published studies focus on algorithm development rather than external validation, clinical implementation, explainability, workflow integration, or regulatory requirements (5, 11). Ongoing challenges, such as breed-specific data bias, limited transparency of “black-box” algorithms, automation bias, and the risk of clinically inappropriate recommendations, continue to hinder the safe adoption of AI (5, 11).

Although several reviews have summarized specific AI applications in veterinary medicine, the literature remains fragmented. Most existing reviews focus on individual application domains or provide narrative overviews without the transparent study selection and reproducible methodology expected of PRISMA-based systematic reviews (3, 11–13). To our knowledge, no recent PRISMA-based systematic survey has comprehensively synthesized AI methodologies, validation strategies, implementation readiness, emerging large language model (LLM) applications, and commercially available veterinary AI systems across multiple domains.

Accordingly, this systematic survey addresses the following research question: *What is the current state of AI applications in veterinary digital health, and what methodological, clinical and implementation challenges remain for their safe integration into veterinary practice?* The objectives are to synthesize current evidence, evaluate AI methodologies and validation strategies, assess methodological quality, identify implementation challenges, and highlight future research priorities to support the ethical and evidence-based integration of AI into veterinary medicine (3, 12, 14, 15).

### 1.1 Background

Veterinary digital health encompasses the application of information and communication technologies to animal healthcare, including eHealth, mobile monitoring, telemedicine, and telehealth (16, 17). These technologies are increasingly integrated with the Internet of Medical Things (IoMT) and AI enabled analytical systems to enhance the delivery, management, accessibility, continuity and quality of veterinary healthcare services (15, 18). Key applications include telemedicine, remote consultations, teletriage, wearable sensors, and AI-assisted diagnostic systems, which together facilitate earlier disease detection, continuous health monitoring, and improved access to veterinary services, especially in underserved and rural areas (18–21).

Within the digital health ecosystem, AI serves as the analytical layer that transforms diverse veterinary data into clinically actionable information. Machine learning (ML) is commonly used for predictive modeling, pattern recognition, and clinical decision support. Deep learning (DL), particularly convolutional neural networks (CNNs), has demonstrated promising performance in medical image classification and disease detection (4, 22, 23). Natural Language Processing (NLP) enables the extraction of clinically relevant information from unstructured veterinary medical records, supporting automated documentation, information retrieval, and clinical decision-making (1, 24). While these methods have demonstrated promising results in controlled research environments, relatively few studies have reported prospective clinical implementation or external validation.

**Figure 1:**
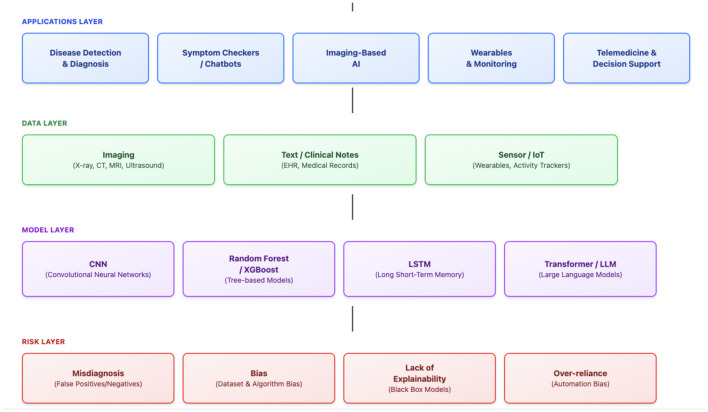
Author-developed conceptual framework summarizing major AI application domains identified through thematic synthesis of the included studies.

Veterinary AI must address significant biological diversity across species and breeds, necessitating species-specific models and validation strategies (10). Model development is further challenged by fragmented veterinary health records, limited standardized datasets, and small sample sizes, which reduce reproducibility and generalizability across clinical settings and animal populations (10). Additionally, veterinary AI operates within a less developed regulatory framework than human healthcare, with limited guidance for AI-based clinical decision-support systems (4, 10, 25). Collectively, these technical, methodological, and regulatory challenges indicate that successful implementation requires not only strong algorithmic performance but also robust validation, methodological transparency, and real-world clinical applicability. An overview of the AI application domains discussed in this review is presented in Figure 1.

## 2 Methodology

**Figure 2:**
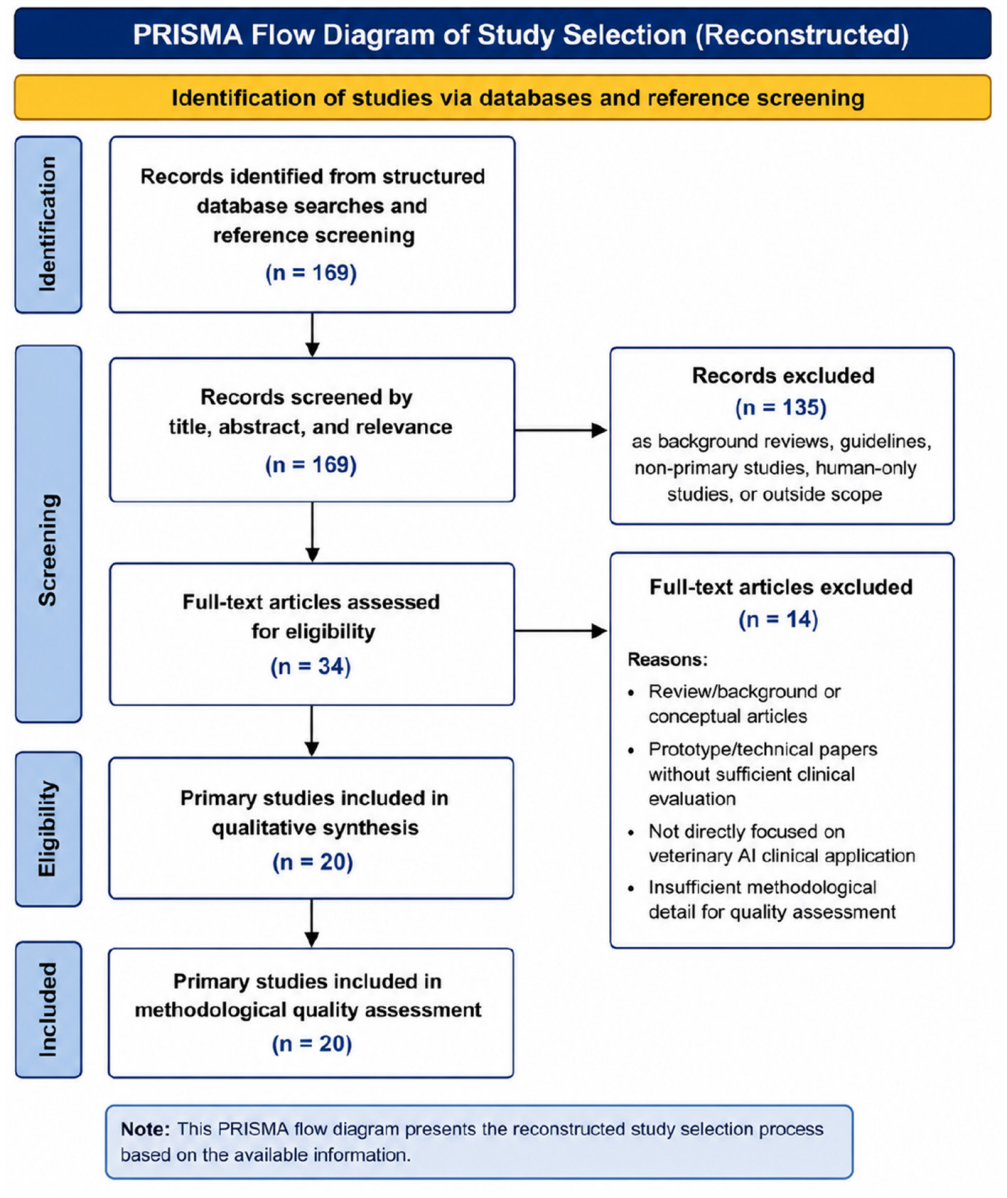
PRISMA 2020 flow diagram illustrating literature identification, screening, eligibility assessment, and study inclusion.

To identify relevant peer-reviewed literature, this systematic survey adhered to PRISMA 2020 guidelines, ensuring a standardized and rigorous review of Artificial Intelligence (AI) applications in veterinary digital health, as well as enhanced transparency and reproducibility (2, 3). A multi-database search was conducted across PubMed, Scopus, Web of Science, and IEEE Xplore to provide comprehensive coverage of clinical, technical, and veterinary research (3, 26). The search strategy utilized Boolean operators with keywords such as “artificial intelligence,” “machine learning,” “deep learning,” “veterinary,” “veterinary medicine,” “animal health,” and “digital health” (3, 13). The temporal scope was limited to articles published between 2013 and 2025 to capture recent advances in AI development, current best practices, and the rapid expansion of sophisticated AI applications in veterinary medicine (3, 26, 27). Complete search strategies for each database are detailed in Supplementary Appendix A. The review process is summarized in the PRISMA 2020 flow diagram (Figure 2).

Eligible studies were peer-reviewed journal articles published in English that addressed the direct application of AI models to animal disease detection, clinical management, diagnostic imaging, behavior monitoring, wearable technologies, telemedicine, livestock health management, or clinical decision support (3, 12). Studies employing only rule-based logic without AI or machine learning components were excluded to maintain data integrity (3). Additional exclusion criteria included studies focused exclusively on human healthcare, meeting abstracts without full-text availability, studies utilizing AI solely for data preprocessing, and non-veterinary applications such as animal models developed exclusively for human health research(3, 26). Publications that misclassified conventional statistical techniques as AI methodologies were also excluded (3). The restriction to English-language publications introduces the potential for language bias, which should be considered when interpreting the findings.

The study selection process was conducted in accordance with the PRISMA 2020 framework (Figure 2). Literature identification, screening, eligibility assessment, and data extraction were performed using predefined inclusion and exclusion criteria. A total of 169 literature resources were identified through structured database searching and reference screening. After title, abstract, and relevance screening, 34 full-text articles were assessed for eligibility. Based on the inclusion and exclusion criteria, 22 studies met the eligibility criteria, comprising 18 primary evidence studies and 4 supporting domain or prototype studies, which were retained to provide methodological and contextual perspectives on emerging AI applications in veterinary digital health.

Study screening, eligibility assessment, and data extraction were performed by a single reviewer using predefined eligibility and data extraction criteria. Because this systematic survey was conducted by a single reviewer, formal inter-reviewer agreement procedures were not applicable. This represents a methodological limitation, as single-reviewer screening may increase the risk of study selection bias and subjective judgment. To minimize the risk, standardized eligibility criteria and a structured data extraction framework were used to promote consistency throughout the review process.

Data extraction was conducted using a standardized framework to ensure consistent evaluation of the included studies. Extracted information comprised publication year, study objective, animal species, clinical application, dataset characteristics, sample size, AI methodology, validation strategy, performance metrics, clinical applicability, reproducibility, and key findings. Because of substantial heterogeneity in study design, animal species, AI methodologies, datasets, and reported outcome measures, findings were synthesized narratively rather than through quantitative meta-analysis.

Author developed conceptual figures were generated through thematic synthesis of the included studies. Following data extraction, studies were grouped according to common application domains, AI methodologies, implementation challenges, and clinical themes. Quantitative figures (e.g., AI model families, animal species, and primary data modalities) were derived directly from the extracted study characteristics, whereas conceptual figures summarize recurring themes identified across the included literature rather than representing results from individual studies.

To strengthen the interpretation of the evidence, the methodological quality of the included studies was assessed using a structured appraisal framework developed for this review. A validated risk-of-bias or quality assessment instrument was not adopted because the included studies represented heterogeneous study designs, including diagnostic imaging, machine learning, wearable technologies, telemedicine applications, clinical decision-support systems, and emerging AI prototype frameworks, for which no single validated appraisal tool was appropriate across all study types. Therefore, a standardized methodological appraisal framework was developed to enable consistent evaluation of reporting quality, methodological transparency, validation practices, reproducibility, and clinical applicability across the included studies. Each study was evaluated across ten methodological domains: objective clarity, dataset description, sample size reporting, AI methodology transparency, validation strategy, external validation, reporting of performance metrics, discussion of bias and generalizability, clinical applicability, and reproducibility. Based on the overall completeness of reporting and methodological rigor, studies were categorized as High, Moderate, or Low methodological quality.

## 3 Results

### 3.1 Characteristics of included studies

**Figure 3:**
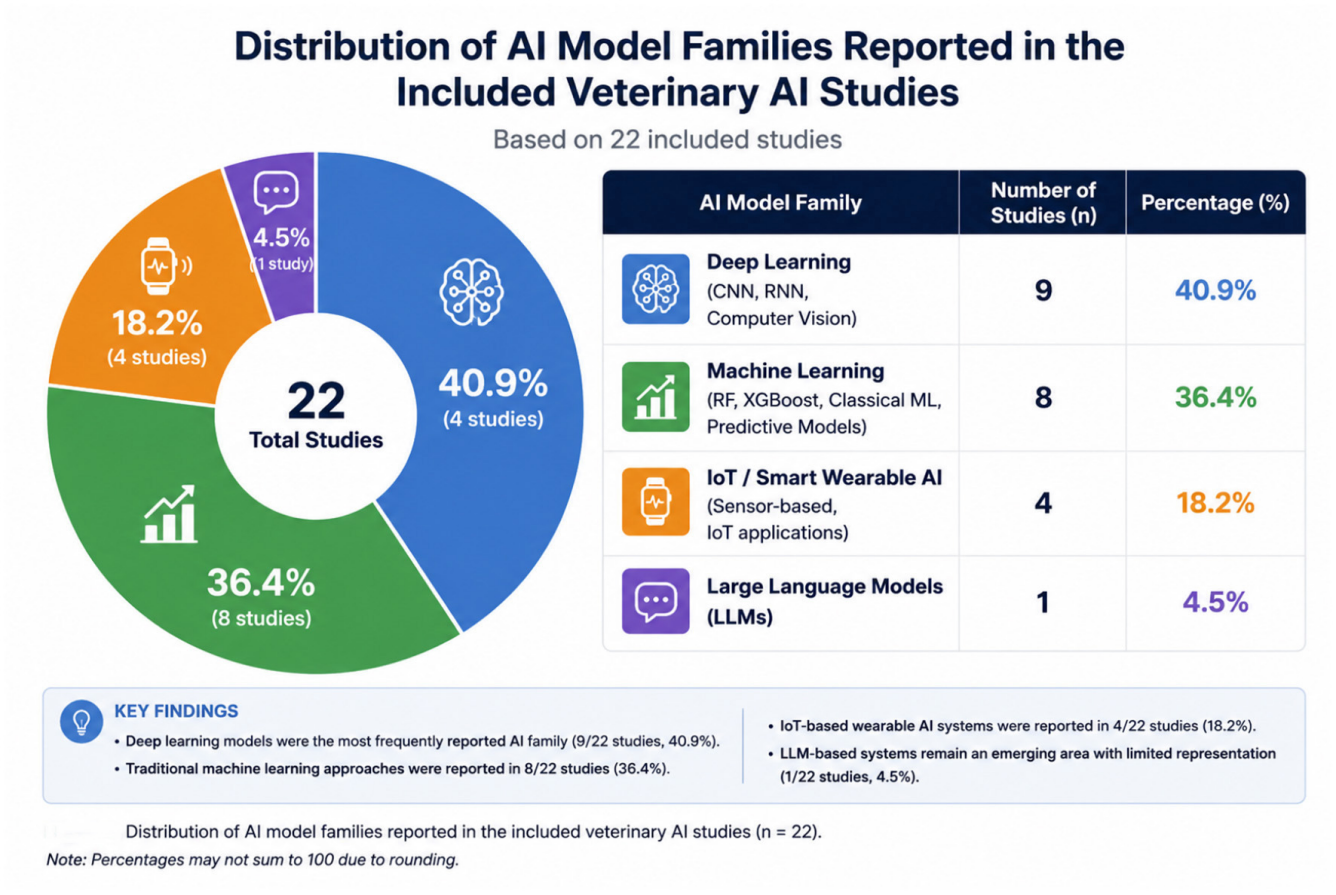
Distribution of AI Model Families reported in the included veterinary AI studies.

**Figure 4:**
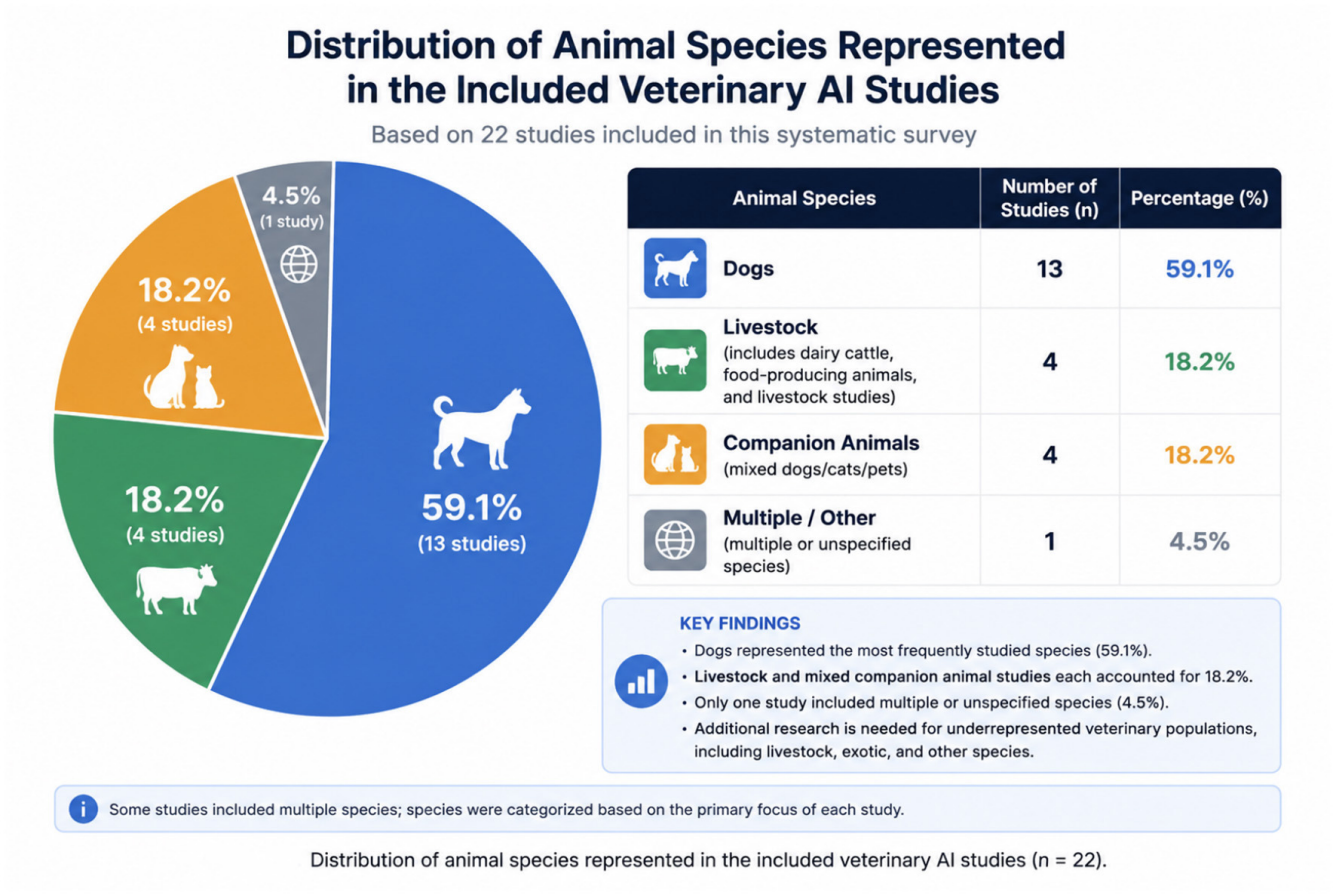
Distribution of animal species represented in the included veterinary AI studies.

A total of 22 studies were included, consisting of 18 primary evidence studies (81.8%) and 4 supporting domain or prototype studies (18.2%). The included literature evaluated AI applications in diagnostic imaging, disease detection and prediction, wearable monitoring, livestock health management, clinical decision support, telemedicine, and emerging large language model (LLM)-based systems. The studies represented diverse veterinary populations, with dogs accounting for the largest proportion of included studies (13/22, 59.1%), followed by livestock-related studies (4/22, 18.2%). Additional studies investigated companion animals, pets, mixed canine-feline populations, and multiple or unspecified species. The included literature employed a range of AI methodologies, including machine learning, deep learning, computer vision, wearable AI systems, and LLM-based approaches. The distribution of AI model families reported across the included studies is presented in Figure 3. Validation approaches included cross-validation, independent test datasets, external validation, prototype validation, and clinical implementation studies. Supplementary Table S1 summarizes the characteristics and methodological quality of the included studies. The distribution of animal species represented across the included studies is shown in Figure 4.

### 3.2 Methodological quality assessment

The quality assessment identified 9 high-quality studies (40.9%), 6 moderate-quality studies (27.3%), and 7 low-quality studies (31.8%). A summary of the methodological quality assessment is presented in Table 1, while the detailed study-level assessment is provided in Supplementary Table S1.

**Table 1:** Summary of the methodological quality assessment of included studies.

Quality	Primary	Supporting	Total
High	9	0	9
Moderate	6	0	6
Low	3	4	7
Total	18	4	22

### 3.3 AI applications in veterinary digital health

#### 3.3.1 Disease detection and diagnosis

**Figure 5:**
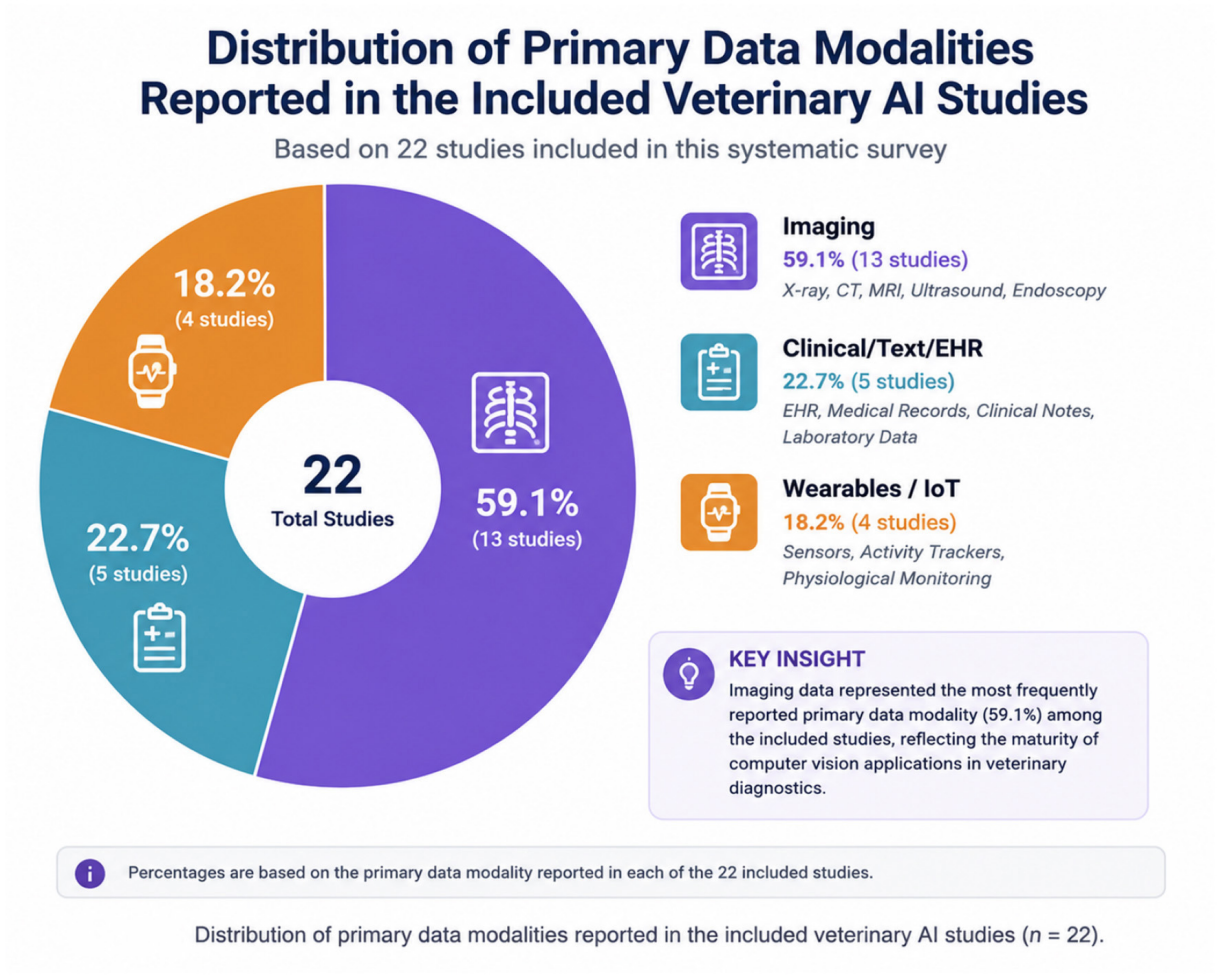
Distribution of primary data modalities identified through systematic data extraction from 22 included veterinary AI studies.

AI-driven Disease Detection and Diagnosis targets critical conditions such as myxomatous mitral valve disease, chronic kidney disease (CKD), hypoadrenocorticism, and various skin disorders (28). Predictive models also predict canine skin diseases (bacterial, fungal), cardiac conditions (MMVD, murmurs), livestock issues such as lameness and lumpy Skin Disease, seizures, and zoonotic outbreaks (1, 9). Reported AI methodologies included Convolutional Neural Networks (CNNs) such as InceptionV3 and ResNet for imaging (3, 5, 9). Random Forest (RF) and RNNs analyze clinical metrics, health records, and sensor data (5, 22). Large Language Models (LLMs) for automated triage (5). Reported performance metrics frequently exceeded 90%, with skin disease detection reaching 89–90%, 96.3% sensitivity for endocrine disease, up to 96.24% accuracy in dermatological classification tasks, and cardiac models achieving 95% accuracy (1, 21). Reported architectures also included Transformers and LLMs for multimodal diagnostic applications (23). The distribution of primary data modalities reported across the included veterinary AI studies is summarized in Figure 5.

#### 3.3.2 Symptom checkers and Chatbots

Reported symptom checker systems included both research frameworks, like AVA, and commercial applications, including Pawprint AI, Ask Vet, HAPPY PET, Ada, K Health, Babylon for human health, Airvet, and VetaFRICA (Ethiopia) (5, 29–31). Reported methodologies included Natural Language Processing (NLP), Bayesian networks, and Large Language Models like GPT-4o (5, 32). These systems extracted symptoms from user input and generated disease or triage recommendations based on predefined knowledge bases or AI models (5, 32–35).

#### 3.3.3 Imaging-based systems

Diagnostic imaging represented the most frequently reported application domain among the included studies. Imaging modalities included radiography, MRI, CT, Ultrasound, digital cytology, and RGB photography (1, 3). Frequently reported architectures included CNNs, ResNet, DenseNet, Inception (V3), EfficientNet, and VGG for image classification tasks (1, 3, 4, 23, 36, 37).

#### 3.3.4 Wearables and monitoring

Reported wearable systems incorporated inertial measurement units (IMUs), biometric sensors, acoustic sensors, pressure sensors, and GPS modules to monitor physiological parameters, activity, movement, feeding behavior, and geolocation (1, 38–43).

#### 3.3.5 Telemedicine and decision support

The included studies reported AI-assisted telemedicine and clinical decision-support systems designed to assist veterinarians in interpreting radiographs, electronic health records, and other clinical information (10, 21, 25, 44–46). These systems were described as supporting diagnostic interpretation and clinical decision-making rather than functioning as autonomous diagnostic tools.

## 4 Discussion

### 4.1 Comparative analysis of ai applications

**Figure 6:**
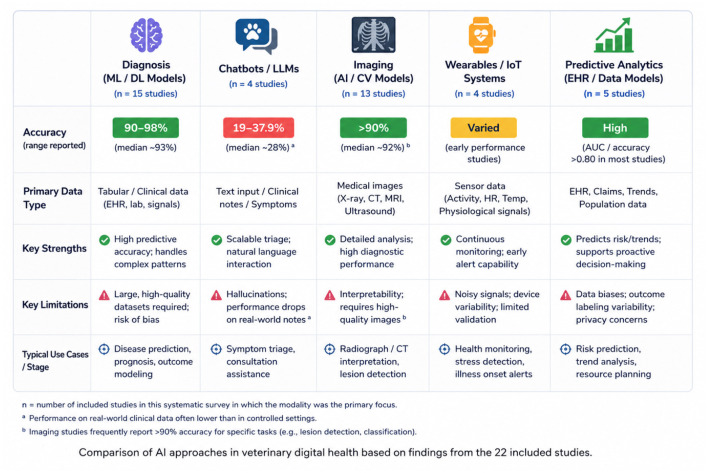
Author developed comparison of AI approaches in veterinary digital health based on the themes identified across the included studies.

The reviewed studies indicate that Deep Learning (DL) and traditional machine learning approaches are the most commonly utilized artificial intelligence methodologies in veterinary digital health, especially for diagnostic imaging, disease prediction, and clinical decision support. Although multiple application domains report promising performance, substantial challenges persist regarding data quality, external validation, and clinical generalizability in real-world settings (47). The effectiveness of AI systems is strongly influenced by the data modality and the chosen model architecture. A comparison of AI approaches in veterinary digital health based on the themes identified across the included studies is presented in Figure 6.

### 4.2 Top performing models

Among the included studies, diagnostic imaging was the application domain most frequently employing Convolutional Neural Networks (CNNs), including ResNet, DenseNet, and VGG architectures are the most prevalent and effective (3, 36). Studies consistently show that these architectures achieve accuracies exceeding 90% for tasks such as detecting cardiomegaly or classifying thoracic lesions (48). For tabular data, such as Electronic Health Records (EHRs) and sensor data, ensemble methods like Random Forest (RF), XGBoost, and CatBoost often achieve strong predictive performance (8, 49). For instance, a stacked ensemble achieved 95% accuracy in predicting fatal safety outcomes using OpenFDA records (49). Emerging research also highlights Vision Transformers (ViTs), which have achieved up to 94.8% accuracy in dermatological classification by capturing global relationships in images better than standard CNNs (50).

For clinical and structured data, Random Forest (RF) often outperforms standalone deep learning models, particularly when datasets are small or class-imbalanced (51). In consultation assistance, Large Language Models (LLMs) such as GPT-4o have demonstrated stable performance, with the AVA framework achieving 91.4% top-3 accuracy in disease prediction in controlled settings (5).

### 4.3 Datasets and generalizability

The majority of veterinary AI research relies on small, localized, or private datasets, often limited to a single institution (4). Public repositories such as Kaggle and the Roboflow universe are frequently used for proof-of-concept studies (50). Large-scale clinical data sources include VetCompass (data from 1800 practices) and SAVSNET (15). Specialized repositories, such as the openFDA veterinary adverse event database (1.28 million reports) and insurance datasets from Fetch, Inc. (2.4 million claims), enable large-scale predictive modeling (8, 49).

However, the biological diversity across species and breeds makes these results difficult to generalize (10, 52). Models trained on single-source data often perpetuate innate biases, failing when applied to patient groups with different ages, breeds, or geographic distributions (36). Large-scale commercial platforms such as SignalRAY, which was trained on images from over 3000 practices across 10 countries to ensure broader robustness (53).

### 4.4 Real world implementation

Although many AI systems report high diagnostic performance under controlled research conditions, translating these models into routine veterinary practice remains a significant challenge (5). Performance frequently declines when evaluated using real-world clinical data, highlighting the importance of external validation and prospective clinical evaluation. For instance, the AVA system's disease prediction accuracy dropped from 91.4% on generated data to between 38.6% and 51.5% on real-world records (5). Some systems, such as SignalPET for radiology, are currently used in veterinary workflows to assist with radiographic interpretation (21). However, symptom checkers intended for lay users generally demonstrate lower diagnostic accuracy (19–37.9%) than veterinarians, raising concerns about potential safety hazards in real-world triage.

**Practical barriers include:**

- **Data quality:** Messy and unstructured clinician notes, inconsistent documentation, and variable image quality (e.g., poor lighting or positioning) hinder model reliability(5).
- **Infrastructure:** Small clinics often lack the GPU resources needed to run sophisticated DL models or LLMs locally (5).
- **Automation bias:** There is a risk that clinicians over-trust AI results, potentially ignoring contrary physical data (1).

### 4.5 Existing applications: technical comparison

Veterinary triage is undergoing a digital transformation as AI-driven applications emerge to streamline clinical workflows and support earlier disease detection and clinical decision-making (10). The landscape of pet symptom triage has evolved from simple search-based inquiries to sophisticated digital platforms, categorized as teletriage, that aim to assess the urgency of clinical signs and inform referral decisions (18, 54). Existing applications range from non-VCPR (Veterinarian-Client-Patient Relationship) services such as Whiskerdocs, ask.vet, and PetCoach, which provide general “teleadvice” by searching databases, to VCPR-compliant platforms such as Airvet, Petzam, and Guardian Vets that connect owners directly with their practitioners (54). These systems primarily target the early stages of consultation, where owners must describe symptoms to determine the urgency of care (5, 7). While many systems remain rule-based, the integration of Artificial Intelligence (AI) is rapidly expanding through text analysis and computer vision (9).

Current triage applications identified in the included studies vary in their architectural complexity, ranging from rule-based systems to advanced Large Language Models (LLMs), and can be divided into five modalities:

**Conversation Chatbots and LLMs:** Research frameworks such as AI Veterinary Assistance (AVA) use LLMs, such as GPT-4, paired with a veterinarian-certified database of 244 diseases and 464 symptoms to extract symptoms from unstructured consultation records and predict probable diseases. While it achieved an impressive 91.4% Top-3 accuracy on generated records, its performance plummeted to 38.6% when applied to real-world, unstructured clinician notes, highlighting a major gap in its real-world adaptability (5).

**HAPPY PET:** A comprehensive mobile app that employs a multi-stage hybrid approach, combining Support Vector Machines (SVM) for image-based detection with AI chatbots for symptom verification. This framework reported high accuracy of up to 98.79% in disease confirmation (35).

**Commercial Consumer Tools:** Commercial applications like Pawprint AI, Ask Vet, Petzey and Pet Pulse have incorporated AI technologies, including Natural Language Processing (NLP), to support owner interactions, remote consultations, and symptom-based guidance (40).

**Diagnostic Imaging Triage:** Specialist tools such as SignalRAY and Vetology assist in triaging radiographic findings (36, 53). SignalRAY achieved 92.7% accuracy for low-ambiguity findings, though it often demonstrated lower sensitivity (57.8%) than board-certified radiologists, suggesting a risk of missing subtle abnormalities (53). Owners increasingly use Convolutional Neural Networks (CNNs) to upload photos of canine skin lesions or eye conditions (3, 9). These models, such as InceptionV3, have demonstrated diagnostic accuracies of 89–98% for specific conditions, such as bacterial or fungal dermatosis (9, 22).

**Wearable Monitoring**: Devices like PetPace and Whistle provide proactive triage by monitoring vital signs, such as heart rate and activity levels, and alerting owners to deviations that may indicate stress or the onset of illness before clinical symptoms are visible (55).

In comparative studies, triage accuracy (determining the level of care needed) typically outperforms diagnostic accuracy (identifying the specific disease) (56). While no digital tool has yet outperformed human practitioners, some, such as the human-focused Ada app, have come close to General Practitioner safety levels, achieving 97% safe triage performance (57). However, general diagnostic accuracy for primary diagnoses remains notably low, ranging between 19% and 37.9% across multiple studies (56, 57). This gap is often exacerbated in veterinary medicine due to species-specific variability and the inability of animal patients to describe their own ailments (3, 58).

### 4.6 Multi-faceted risks of AI triage

The integration of AI into triage introduces significant clinical, ethical, legal, undertriage, and overtriage risks.

#### 4.6.1 Clinical hallucinations

Complex deep learning models are prone to hallucinations, in which they detect patterns in random noise or interpret nonexistent features in medical images. In LLMs, this can lead to unsafe treatment suggestions or the fabrication of scientific references (10).

#### 4.6.2 Automation and lay interpretation bias

There is a profound risk of automation bias, in which clinicians may favor machine-generated decisions over their own professional judgment. For pet owners, lay interpretation bias can lead to cyberchondria, in which owners delay essential care or mismanage cases based on incorrect digital guidance (10).

#### 4.6.3 The black box and trust

Most advanced models are opaque, making it difficult for clinicians to reconstruct the logic behind a triage score (5). This lack of Explainable AI (XAI) undermines the Veterinary-Client-Patient Relationship (VCPR) and makes informed consent difficult to obtain (10).

#### 4.6.4 Dataset and generalizability gaps

Veterinary data is often scattered across silos and lacks the standardization of human medicine (10, 52). Models trained on single-institution data often perpetuate innate biases related to breed, age, or geographic distribution (4).

#### 4.6.5 Undertriage

This represents the greatest safety hazard, in which the system fails to recognize a life-threatening condition, leading the owner to delay necessary care (11). Examples in human medicine include AI delaying care for strokes, a risk that is equally critical for veterinary emergencies like gastric torsion or heart failure (11).

#### 4.6.6 Overtriage

**Figure 7:**
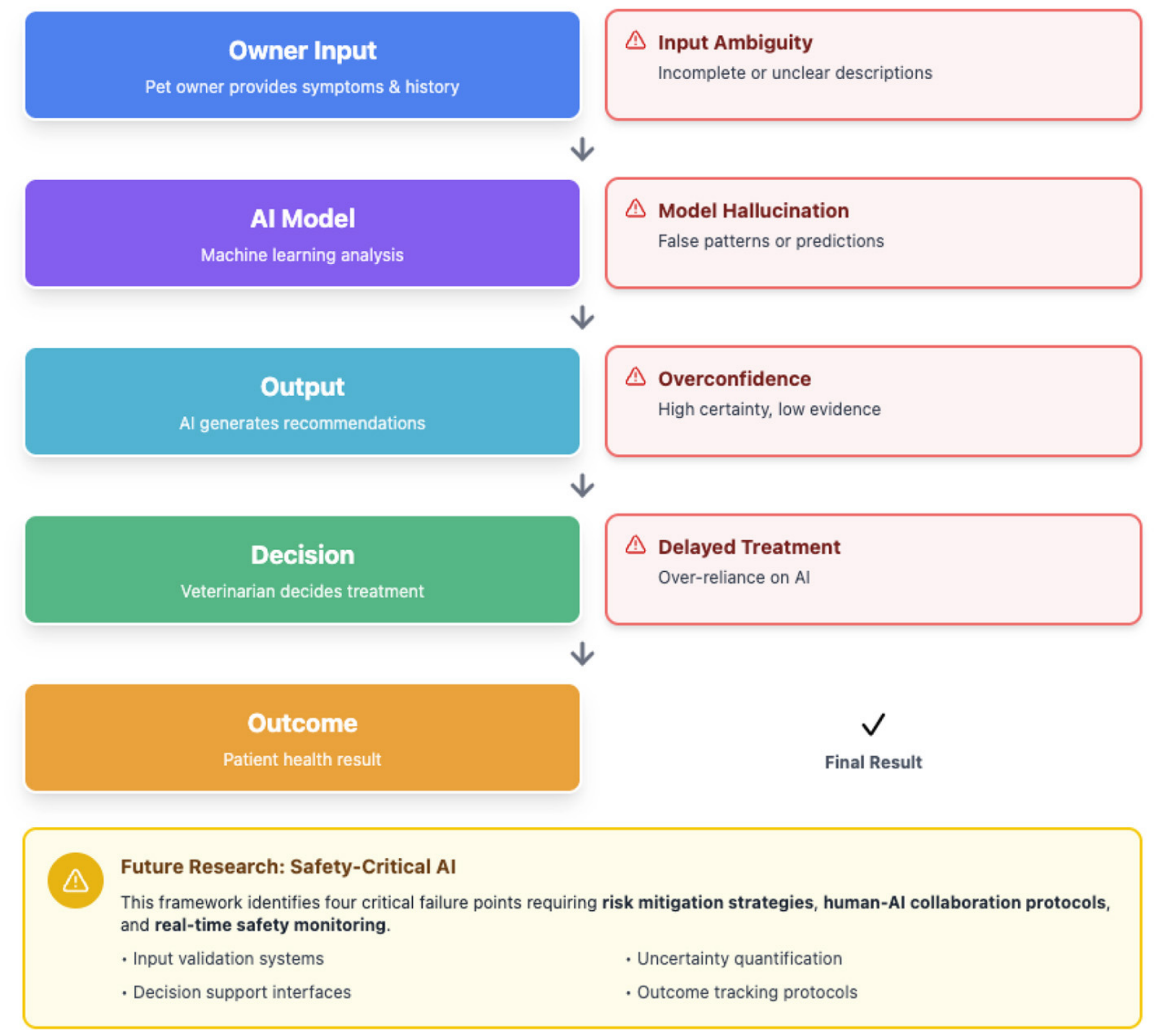
Author developed conceptual framework illustrating potential sources of error in AI-assisted veterinary decision systems based on the thematic synthesis of the included studies.

Often a result of risk-averse programming, it recommends higher levels of care than necessary, leading to unnecessary ER visits and increased strain on the healthcare system (59). The principal sources of error associated with AI-assisted veterinary decision systems are summarized in Figure 7.

## 5 Legal, regulatory, and ethical gaps

**Figure 8:**
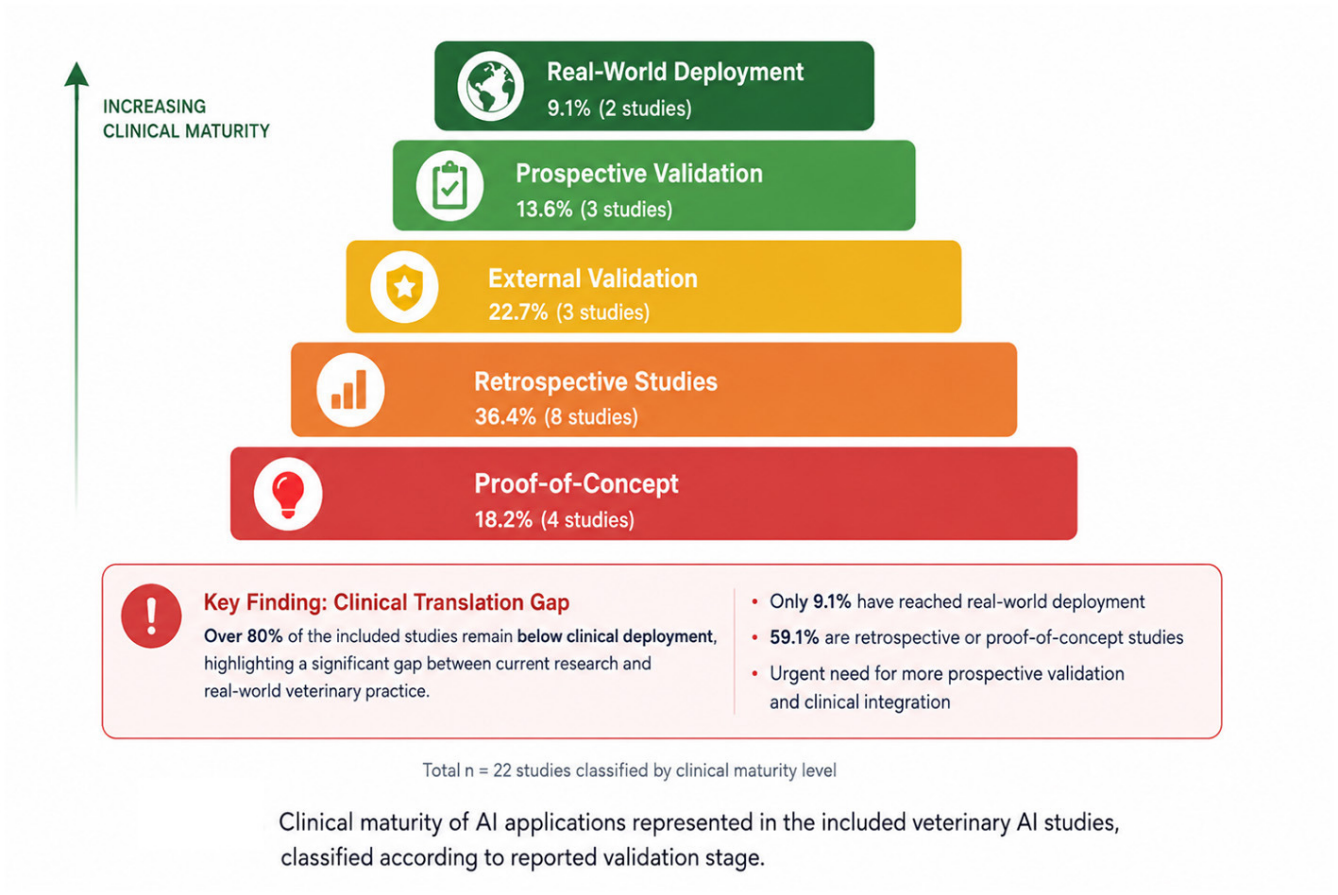
Clinical maturity of AI applications represented in the included veterinary studies (*n* = 22), classified according to the reported validation and deployment stage.

In North America, there are currently no federal premarket approval requirements for veterinary AI software, creating a regulatory vacuum in which the responsibility for appropriate use rests entirely with the veterinarian (10, 25). Unlike human medicine, where tools must pass rigorous FDA or EMA approval, veterinary AI products often reach the market without peer-reviewed validation(10). Consequently, the liability for errors remains solely with the individual veterinarian, who must balance the efficiency of AI assistants against the fundamental duty to first do no harm (10). Furthermore, triage relies heavily on owner-reported history, which is frequently incomplete or misinterpreted, leading to potential diagnostic missteps (18, 58). Effective implementation will require new governance standards that prioritize human-in-the-loop oversight to ensure AI remains a supportive tool rather than an autonomous decision-maker (60). Ultimately, while these tools offer the potential to increase accessibility in veterinary deserts, they are currently viewed as decision-support tools that must augment, rather than replace, professional clinical judgment (3). The clinical maturity of AI applications represented across the included veterinary studies is summarized in Figure 8.

## 6 Gap analysis

The landscape of pet symptom triage has shifted from simple Google searches to sophisticated Artificial Intelligence (AI applications) (11, 18). Research frameworks such as AI Veterinary Assistance (AVA) and commercially available applications, including Pawprint AI, Ask Vet, Whiskerdocs and PetCoach, illustrate the expanding use of AI for symptom extraction, disease prediction, owner guidance, and teletriage (5, 18). Collectively, these technologies have been propose as decision-support tools that may improve access to veterinary guidance, although evidence regarding their real-world clinical effectiveness remains limited.

### 6.1 The data and species disparity gap

A primary research gap is the extreme bias toward species and breeds in the training data. Among the included studies, dogs represented the most frequently investigated species (13/22, 59.1%), followed by livestock-related studies (4/22, 18.2%) and mixed companion animal studies (4/22, 18.2%). Only one study (4.5%) investigated multiple or unspecified species, highlighting the comparatively limited representation of livestock, equine, exotic, and other veterinary populations. Furthermore, many models lack breed-specific morphological knowledge. For instance, a diagnostic tool trained on standard canine skull radiographs may misinterpret the unique anatomy of an English Bulldog, leading to misdiagnosis (25). This is exacerbated by fragmented data repositories that operate in vendor-specific silos, prohibiting the interoperable exchange of medical records necessary to train robust, generalized models (15).

### 6.2 The real-world validation and performance gap

There is a significant disconnect between lab performance and real-world clinical utility (11). High accuracy has been reported in controlled research settings and in live clinical environments. Most existing evaluations rely on simulated patient vignettes rather than real-patient data, which fail to capture the complexity and nuance of actual clinical presentations, such as messy documentation, inconsistent data entry, and diverse patient demographics (61).

While some research models, such as the AVA (AI Veterinary Assistance) framework, claim 91.4% accuracy in disease prediction on organized, generated records, the accuracy plummeted to 38.6% when applied to real-world, unstructured electronic health records (EHR) (5). Systematic reviews of consumer-facing symptom checkers report primary diagnostic accuracy ranging from 19% to 37.9% (56). This performance gap introduces the severe risk of undertriage, where a life-threatening condition like gastric torsion or a mini stroke is classified as non-urgent, leading owners to delay critical care (11). Conversely, overtriage, recommending emergency care for minor ailments, strains healthcare resources and increases owner anxiety (11, 56).

### 6.3 The infrastructure and scalability gap

Unlike large human medical institutions, most veterinary clinics operate on a small scale and lack the computational infrastructure (e.g., dedicated servers or GPUs) needed to run sophisticated deep learning models (LLMs) locally (5). Consequently, even highly effective research models often remain undeployable for the average practitioner due to high operational costs and space constraints (5). Furthermore, veterinary data is often fragmented into silos, preventing the creation of large standardized datasets necessary for training generalizable models across multiple species and breeds (62).

### 6.4 The technical, explainability, and trust gap

Most high-performing triage systems use architectures such as convolutional neural networks (CNNs) that operate as “black boxes,” offering little transparency into how they arrive at a specific diagnosis or triage score (5, 63). This lack of Explainable AI (XAI) prevents veterinarians from acting on AI recommendations when the logic is unclear, especially in life-critical scenarios or when contradictory clinical evidence exists (25, 62, 64). While some frameworks are beginning to incorporate Explainable AI (XAI) to show intermediate reasoning steps, such as which specific symptoms triggered a disease candidate, very few veterinary studies have technically addressed how to make model reasoning transparent to the user (3, 5).

### 6.5 The sequential reasoning and uncertainty gap

Prevailing AI triage approaches often assume that complete data (including vital signs and laboratory results) is available before generating a prediction (65). This fails to mirror the sequential nature of clinical workflows, where a triage decision must often be made under conditions of high uncertainty, using only an owner's description of symptoms (65). Furthermore, current systems often lack clear mechanisms to handle uncertainty or failure in symptom extraction, potentially leading to clinical hallucinations in which the AI provides an unsafe or incorrect treatment recommendation without a warning flag (5).

### 6.6 Clinical, regulatory, and accountability risks

Perhaps the most critical gap is the lack of regulatory oversight. In North America, there are currently no federal premarket approval requirements for veterinary AI software (Software as a Medical Device) (25). Unlike human medicine, where AI tools are subject to rigorous Class II-IV risk classification, veterinary tools are often treated as unregulated or low-risk (Class I) (66). This regulatory vacuum leaves the legal and ethical liability for any AI-driven misdiagnosis entirely with the individual veterinarian, who must justify medical decisions without a standardized framework for error reporting or clinical validation (25). This lack of oversight exacerbates existing risks:

- **Automation Bias:** Practitioners may over-rely on automated scores, ignoring contrary physical data or their own clinical judgment (1, 10).
- **Lay Interpretation Bias:** Pet owners may misinterpret triage advice, leading them to delay essential care or to attempt inappropriate home remedies based on a chatbot's suggestions (67, 68).
- **Algorithmic Bias:** Models trained on single-institution data often perpetuate biases related to breed, age, or geographic distribution, limiting their safety for the broader pet population (62, 69).

To bridge these gaps, future development must move toward risk-aware AI that incorporates uncertainty quantification and human-in-the-loop oversight (65, 70). The veterinary profession requires integrated frameworks that dismantle data silos and establish standardized benchmarking protocols that measure real-world clinical utility rather than just retrospective accuracy, to ensure that these digital tools enhance, rather than compromise, animal welfare (3, 15, 62).

## 7 Key challenges and limitations

The integration of AI into veterinary digital health is hindered by several systemic challenges, primarily data limitations, validation gaps, and the inherent opacity of complex models.

### 7.1 Data scarcity, silos, and the ‘DRIP' problem

A primary challenge is that veterinary medicine is often “data rich but information poor” (DRIP) (4). Unlike human healthcare, which benefits from standardized datasets, veterinary data is typically scattered in isolated silos within individual clinics, making it difficult to collate the large-scale, high-quality datasets required for training robust AI (10). Datasets are typically small and localized, and existing records are often unstructured, consisting of memo-style notes and abbreviations, and lack consistency (5). Among the included studies, dogs represented the most frequently investigated species (13/22, 59.1%), followed by livestock-related studies (4/22, 18.2%) and mixed companion animal studies (4/22, 18.2%). Only one study (4.5%) investigated multiple or unspecified species, highlighting the comparatively limited representation of livestock, equine, exotic, and other veterinary populations. This bias frequently leads to overfitting, where a model performs well on training data but fails to generalize to new patient populations. For instance, models trained on small-breed dogs (< 15 kg) are often optimized for their specific morphologies and may misdiagnose larger breeds (25, 71). Another example: an AI never trained on brachycephalic anatomy may misdiagnose a standard radiograph of an English Bulldog (25).

In diagnostic imaging, technical variations such as suboptimal patient positioning or poor lighting significantly degrade model performance compared to idealized research conditions (1, 4). Furthermore, much of this data is sourced from secondary insurance claims or referral hospitals, which may contain inaccurate owner-reported breeds or represent only the most complex clinical cases rather than the general population (8).

### 7.2 Real-world validation

There is a profound disconnect between the accuracy of offline research and the clinical utility of online research (61). For example, the AVA framework achieved a disease prediction accuracy of 91.4% on generated records, yet its performance plummeted to 38.6% when applied to real-world, unstructured clinician notes (5). Most existing studies rely on clinical vignettes that do not capture the unique challenges of real-world care, such as messy documentation, inconsistent owner descriptions, and biological diversity across species and breeds (36).

### 7.3 The black box and the interpretability crisis

Most high-performing architectures, such as Deep Learning (DL) and Convolutional Neural Networks (CNNs), operate as black boxes (5). This lack of transparency is a major barrier to adoption; veterinarians are ethically and legally hesitant to trust or act on recommendations they cannot interpret (5). Developers face a persistent trade-off between model accuracy and interpretability, where the most precise systems are often the least understandable to clinicians and pet owners (72).

### 7.4 Multi-symptom reasoning and uncertainty

Current AI triage models frequently assume that complete clinical data (e.g., vital signs and lab results) is available before generating a prediction (65). This fails to mirror the sequential nature of triage, where prioritization decisions must often be made under conditions of high uncertainty, using only an owner's initial description of symptoms (65). Most existing veterinary AI tools are narrow in scope, focusing on single diseases or specific procedures rather than holistic patient assessment (5, 61). While emerging frameworks like AVA attempt to extract multiple symptoms from consultation notes, most systems still struggle with complex context and co-occurring infections (5). Additionally, many systems lack clear mechanisms to handle uncertainty or extraction failures, potentially leading to clinical hallucinations in which the AI provides unsafe or fabricated treatment suggestions without a warning flag (5).

### 7.5 Pet owner usability and risks

While pet owners seek 24/7 digital guidance, current tools often lack user-friendliness and affordability (7, 40). Many current mobile applications rely on rigid input requirements and preset symptom lists, which prevent owners from describing the nuances, frequency, or complexity of coexisting symptoms (29, 66). Owners frequently encounter navigation challenges, confusing medical jargon, and impenetrable interfaces (66). Wearable sensors also suffer from hardware limitations, including short battery life, a lack of user-friendly recalibration protocols, and the provision of overwhelming volumes of data that owners may not have the training to interpret correctly, making them less practical for rural or low-resource settings (43, 73).

Additionally, symptom checkers frequently suffer from low accuracy, posing a risk of lay interpretation bias, where owners might inappropriately self-diagnose or delay essential veterinary care based on a machine's suggestion (67). The ability of these systems to support multi-symptom reasoning is inconsistent. Advanced virtual triage engines use Bayesian networks to compute probabilities and dynamically adapt questions based on prior answers (33).

Finally, systems often cause workflow disruptions, requiring clinicians to spend extra time calling owners to clarify inaccurate or unfocused reports (74). Users frequently report that these tools lack the subtlety of a physical exam and fail to integrate the animal's full medical history (66).

## 8 Research gaps

The current research landscape in veterinary digital health reveals several critical gaps that hinder its full clinical integration. The absence of a unified triage system is primarily due to fragmented data repositories operating in vendor-specific silos, which prohibit the exchange of information (15). There is no consensus on search terms or standardized benchmarking protocols to evaluate these systems' accuracy across different clinical contexts (66).

Risk scoring remains limited because most predictive models are species- or breed-specific, often optimized for small-breed dogs while failing to account for the extreme morphological variability of larger animals or exotic species (71). Currently, there is no consistent approach for predicting multiple disease categories from broad datasets, such as insurance claims (8).

Real-time decision support is constrained by a profound lack of real-world validation; most of the literature focuses on technical evaluations in controlled lab settings rather than actual clinical practice (46, 75). Additionally, these systems often cause workflow disruptions and increased administrative burdens, making them difficult to integrate at the point of care.

Explainability is a major barrier, as many high-performing models are black boxes with opaque inner workings that do not disclose their reasoning (25, 63). Technical interpretability, understanding how specific input features contribute to a prediction, remains an open research question that very few veterinary studies have technically addressed (3, 63).

Finally, longitudinal tracking is underdeveloped because current monitoring tools are often disconnected from Electronic Health Records (EHRs) (15). Traditional methods like physical tags are insufficient for continuous, quantitative data recording needed to detect long-term health trends or implement preventive measures (75).

## 9 Future directions

The future of veterinary digital health centers on transitioning AI from black-box diagnostic tools to transparent, integrated companions for care. Future systems must prioritize risk-aware AI by incorporating uncertainty modeling and confidence scores to identify borderline cases. This approach helps mitigate automation bias and allows veterinarians to justify clinical decisions with their own professional judgment.

The next generation of diagnostics will leverage multi-modal inputs, moving beyond single-modality models. By fusing data from wearable sensors (activity, heart rate), medical imaging (radiographs, ultrasound), and Electronic Health Records (EHRs), AI can provide a holistic view of animal welfare. Multimodal Large Language Models (LLMs) are already demonstrating the ability to synthesize visual symptoms with textural clinical reasoning.

To enhance owner compliance, Explainable AI (XAI) is essential. Future interfaces must provide transparent reasoning, explaining why specific questions are asked during triage and how symptoms contribute to a predicted result. Moving toward interactive feedback loops will allow users to query model reasoning and provide contextual clinical insights, turning AI into a collaborative dialogue rather than a passive output.

Finally, integrated mobile systems will bridge vendor-specific data silos to ensure longitudinal health tracking. Leveraging Edge AI for local, real-time processing of signals, such as detecting stress or seizures on wearable devices, will enable immediate emergency responses. Seamless interoperability between mobile applications and Practice Information Management Systems (PIMS) will ensure that real-time data is always accessible at the point of care.

## 10 Limitations of this systematic survey

This systematic survey has several methodological limitations that should be considered when interpreting the findings. First, study screening, eligibility assessment, and data extraction were performed by a single reviewer, which may have increased the risk of study selection bias, even with predefined eligibility criteria and a standardized data extraction framework. Second, methodological quality was evaluated using a structured appraisal framework specifically developed for this review, as no single validated risk-of-bias instrument was appropriate for the heterogeneous study designs included, encompassing diagnostic imaging, machine learning, wearable technologies, telemedicine applications, and prototype AI systems. Consequently, the quality assessment should be interpreted as an evaluation of methodological reporting and transparency rather than a formal risk-of-bias assessment. Finally, the review was limited to English-language peer-reviewed publications, which may have introduced language and publication bias.

## 11 Conclusion

This systematic survey suggests that Artificial Intelligence (AI) has considerable potential to support veterinary digital health, particularly in diagnostic imaging, clinical decision support, wearable monitoring, and precision livestock applications. Across the included studies, AI methods frequently achieved high performance under controlled research conditions, especially for imaging-based tasks. However, the evidence synthesized in this review also indicates that widespread clinical implementation remains constrained by fragmented datasets, species specific biological diversity, limited external and prospective validation, and the lack of standardized evaluation frameworks.

The methodological quality assessment further highlights that although several studies reported robust datasets, transparent methodologies, and clinically meaningful validation, many emerging applications remain at the prototype or early validation stage. Consequently, current evidence supports the use of AI as a clinical decision-support tool rather than an autonomous decision-maker.However, these conclusions should be interpreted in the context of the heterogeneous study designs and the variable methodological quality of the available evidence.

Future research should prioritize standardized datasets, prospective multi-center validation, explainable AI (XAI), and governance frameworks that facilitate safe, transparent, and clinically reliable deployment. Continued interdisciplinary collaboration among veterinarians, AI researchers, regulatory organizations, and industry may facilitate the development of future AI systems improve clinical practice while preserving professional judgment, patient safety, and animal welfare.

## Data Availability

The original contributions presented in the study are included in the article/Supplementary material, further inquiries can be directed to the corresponding author.
