# Supplementary material for: AI applications in veterinary digital health: a systematic survey
Source: Front Vet Sci. 2026 Jul 30;13:1853395. doi: 10.3389/fvets.2026.1853395 (PMC13467759; doi:10.3389/fvets.2026.1853395)
Supplement: Supplementary file 1 [file Data_Sheet_1.pdf]

AI Applications in Veterinary Digital Health: A Systematic Survey

1 SUPPLEMENTARY APPENDIX A. DATABASE SEARCH STRATEGY

1.1 Literature Search Strategy

A comprehensive literature search was conducted to identify studies investigating Artificial Intelligence (AI) applications in veterinary digital health. Electronic database searches were performed in PubMed, Scopus, Web of Science, and IEEE Xplore. Searches were limited to peer-reviewed journal articles published in English between January 2013 and March 2025. The search strategy combined controlled vocabulary (where applicable) with free-text keywords using Boolean operators. Search terms were adapted slightly to accommodate the syntax requirements of each database while maintaining equivalent search concepts.

1.2 PubMed

(”artificial intelligence” OR ”machine learning” OR ”deep learning” OR ”neural network*” OR ”convolutional neural network*” OR CNN OR ”large language model*” OR LLM OR ”natural language processing”)

AND

(”veterinary” OR ”veterinary medicine” OR ”animal health” OR livestock OR ”companion animal*” OR canine OR dog OR feline OR cat OR equine OR horse OR bovine OR cattle)

AND

(”digital health” OR telemedicine OR telehealth OR wearable* OR monitoring OR ”diagnostic imaging” OR radiology OR cytology OR ”clinical decision support”)

1.3 Scopus

TITLE-ABS-KEY

(”artificial intelligence” OR ”machine learning” OR ”deep learning” OR ”neural network*” OR CNN OR ”large language model*” OR LLM)

AND

(veterinary OR ”veterinary medicine” OR ”animal health” OR livestock OR ”companion animal*”)

AND

(”digital health” OR telemedicine OR telehealth OR wearable* OR monitoring OR ”clinical decision support” OR ”diagnostic imaging”)

1.4 Web of Science

TS =

(”artificial intelligence” OR ”machine learning” OR ”deep learning” OR ”large language model*” OR LLM OR ”natural language processing”)

AND

(veterinary OR ”veterinary medicine” OR ”animal health”)

AND

(”digital health” OR telemedicine OR telehealth OR wearable* OR monitoring OR ”clinical decision support” OR ”diagnostic imaging”)

1.5 IEEE Xplore

(”Artificial Intelligence” OR ”Machine Learning” OR ”Deep Learning” OR ”Neural Networks” OR ”Large Language Models”)

AND

(Veterinary OR ”Animal Health”)

AND

(”Digital Health” OR Telemedicine OR Telehealth OR Wearable OR Monitoring OR ”Clinical Decision Support” OR ”Diagnostic Imaging”)

1.6 Eligibility Criteria

Studies were eligible for inclusion if they:

- Were published between 2013 and 2025;
- Were published in English;
- Were peer-reviewed journal articles;
- Investigated AI applications in veterinary medicine or animal health;
- Addressed diagnostic imaging, disease prediction, wearable monitoring, livestock health management, telemedicine, or clinical decision support;
- Reported original research involving AI, machine learning, deep learning, or related computational methodologies.

1.7 Exclusion Criteria

Studies were excluded if they:

- Focused exclusively on human healthcare without veterinary relevance;
- Did not implement Artificial Intelligence or Machine Learning methods;
- Consisted solely of rule-based systems without AI components;
- Were conference abstracts, editorials, letters, or opinion articles without full methodological details;
- Used AI solely for data preprocessing without direct veterinary clinical application;
- Represented animal models developed exclusively for human biomedical research;
- Misclassified conventional statistical methods as AI.

1.8 Screening and Study Selection

Literature screening followed the PRISMA 2020 reporting framework. Records were screened by title, abstract, and full-text eligibility according to predefined inclusion and exclusion criteria. The final qualitative synthesis included 22 studies, comprising:

- 18 primary evidence studies, which formed the basis of the systematic evidence synthesis.
- 4 supporting domain/prototype studies, retained to provide methodological and contextual perspectives on emerging AI applications in veterinary digital health.

The complete study selection process is illustrated in Figure 1 of the main manuscript.
