# Supplementary material for: AI applications in veterinary digital health: a systematic survey
Source: Front Vet Sci. 2026 Jul 30;13:1853395. doi: 10.3389/fvets.2026.1853395 (PMC13467759; doi:10.3389/fvets.2026.1853395)
Supplement: Supplementary file 2 [file Data_Sheet_2.pdf]

AI Applications in Veterinary Digital Health: A Systematic Survey

July 1, 2026

1 Supplementary Table S1.A — Study characteristics

Table 1: Supplementary Table S1.A Study characteristics

Study	Category	AI Domain	Species	Clinical Problem	Dataset	AI Method
McDonald et al. (2024)	Peer-reviewed primary veterinary AI diagnostic study with canine clinical dataset and reported validation/performance.	Cardiology / digital auscultation	Dogs	Heart murmur grading and staging preclinical MMVD	756 dogs from UK referral centers; electronic stethoscope recordings with cardiologist and echocardiography reference assessment	Recurrent neural network transferred/fine-tuned from human heart sound detection
Jin et al. (2025)	Primary veterinary AI decision-support study using LLM-based symptom extraction, disease prediction, and consultation assistance.	Clinical decision support / LLM triage	Companion animals (disease-symptom veterinary database; real-world consultation records)	Symptom extraction, probable disease prediction, and next-question recommendation during veterinary consultations.	Veterinarian-certified database of 244 diseases and 464 symptoms; generated consultation records and real-world consultation records.	Large language model, in-context learning, disease-symptom matching, TabTransformer-style/baseline comparisons
Arockiaraj et al. (2025)	Relevant AI/IoT veterinary digital health prototype, but evidence appears conceptual/prototype-level with limited clinical validation.	Livestock IoT monitoring	Livestock	Real-time health monitoring, anomaly detection, disease prediction, and location tracking.	Prototype sensor data; exact sample size not clearly reported in extracted text.	IoT sensors, ESP32, cloud analytics, ML-based pattern/anomaly detection
Jana et al. (2025)	Primary animal health monitoring study using sensor data and explainable ML in dairy cattle.	Wearable/sensor monitoring; explainable AI	Dairy cattle/cows	Monitoring cattle health/activity and supporting yield/health management.	3-axis accelerometer sensor data from dairy cattle; exact sample size to be verified from full text.	Machine learning classifiers with explainability methods

Study	Category	AI Domain	Species	Clinical Problem	Dataset	AI Method
Jarkoff et al. (2023)	Primary validation study of wearable digital health monitoring in dogs with large-scale dataset.	Wearable monitoring / vital signs	Dogs	Remote heart rate and breathing rate estimation.	Large-scale smart collar dataset; exact number of dogs/measurements to verify from full text.	Smart collar predictive algorithms for heart and breathing rates.
Hwang et al. (2022)	Primary computer vision/deep learning veterinary diagnostic study.	Dermatology / image classification	Dogs	Classification of canine skin diseases using multispectral images.	Multispectral dog skin images; sample size to verify from full text.	Deep learning image classifier
Panigrahy et al. (2025)	Candidate primary comparative AI study for animal disease prediction, but dataset and veterinary specificity require careful interpretation.	Animal disease prediction / health risk assessment	Multiple/unspecified animal datasets	Animal disease prediction and health risk assessment.	Benchmark or public datasets; exact dataset/source to verify.	Comparative ML and DL algorithms
Ndiaye et al. (2025)	Primary comparative diagnostic imaging study directly evaluating commercial AI against veterinary radiologists.	Radiology / commercial AI diagnostic support	Dogs and cats	Radiographic interpretation of canine and feline studies.	Canine and feline radiographic studies; commercial AI software compared with veterinary radiologists.	Commercial AI radiology software
Görisch (2020)	Primary validation study comparing veterinary wearable output with Holter ECG reference.	Wearable monitoring / cardiophysiology	Dogs	Heart rate and heart rate variability measurement.	PetPace smart collar and Holter-ECG measurements; sample size to verify from full text.	Smart collar signal-processing/predictive measurement system
Zhao et al. (2025)	Primary veterinary diagnostic imaging study using deep learning on canine CT images.	Diagnostic imaging / CT	Dogs	Classification of canine middle ear disease status.	Canine computed tomography images; sample size to verify from full text.	Deep learning image classifier
Bhogapurapu et al. (2025)	Primary/development study applying DL to canine skin disease detection, but evidence quality requires verification.	Dermatology / image classification	Dogs	Canine skin disease detection.	Dog skin disease images; exact source/sample size to verify.	Deep learning image classifier
Chinnam et al. (2025)	Primary/development study for canine skin problem detection; may overlap with other dog-skin DL studies	Dermatology / image classification	Dogs	Detection/classification of canine skin problems.	Dog skin problem image dataset; exact source/sample size to verify.	Deep learning/CNN

Study	Category	AI Domain	Species	Clinical Problem	Dataset	AI Method
Turner, Patel (2023)	Relevant livestock digital-health prototype, but limited evidence and AI validation unclear.	Livestock IoT monitoring	Livestock	Health and productivity monitoring.	Prototype/system dataset; exact sample size unclear.	IoT monitoring with analytics; AI/ML details limited
Kim et al. (2023)	Peer-reviewed primary veterinary EHR-based ML risk prediction study.	Predictive modeling / EHR	Dogs	Canine MMVD risk prediction.	Electronic health record data for dogs; sample size to verify from full text.	Machine learning risk prediction models
Biswas (2019)	Relevant computer-vision pet health case study, but evidence appears prototype/case-study level.	Computer vision / pet health diagnosis	Pets	Media/image analysis for pet health diagnosis.	Case-study media/image data; exact source/size unclear.	Computer vision media analysis engine
Buric, Ivasic-Kos (2024)	Primary computer vision veterinary study focused on canine ophthalmology.	Ophthalmology / computer vision	Dogs	Canine ocular disease/structure analysis.	Canine ophthalmology images; exact dataset/sample size to verify.	Computer vision/deep learning, including segmentation/classification approaches
Koo et al. (2021)	Primary veterinary oncology ML outcome prediction study using canine lymphoma patient data.	Oncology / outcome prediction	Dogs	Prediction of dynamic clinical outcomes/survival during chemotherapy for canine lymphoma.	242 canine lymphoma patients treated with (L)-CHOP chemotherapy using three data types.	Machine learning models integrating multiple data modalities
Debes et al. (2023)	Large-scale primary ML study using insurance claims data to predict canine disease categories.	Predictive modeling / insurance claims	Dogs	Prediction of future canine health outcomes/disease categories.	785,565 insured dogs from US/Canada over 17 years; 270,203 long-tenure dogs used for model training.	Multiple machine learning approaches with feature engineering
Sholehrasa et al. (2025)	Primary veterinary safety/pharmacovigilance ML/XAI study using real-world FDA adverse event data.	Pharmacovigilance / safety prediction / XAI	Food-producing animals/multiple veterinary species	Prediction of death vs recovery and veterinary safety/residue-related outcomes.	OpenFDA Center for Veterinary Medicine adverse event reports and physicochemical properties; exact sample size to verify.	Predictive modeling with explainable AI
Dourson et al. (2023)	Primary deep learning study for canine ECG classification.	Cardiology / ECG classification	Dogs	Classification of canine ECG as normal or abnormal.	Canine ECG sequences converted into 8-second Lead II sequences; sample size to verify from full text.	Deep CNN with continuous wavelet transform and random augmentation policy
Wang (2024)	Relevant wearable pet health prototype/validation concept, but study quality and peer-reviewed rigor appear limited.	Pet wearable monitoring	Pets	Pet health/safety monitoring and owner feedback.	Six-month user/owner monitoring and feedback; exact sample size unclear.	Smart wearable system with health monitoring/analytics

Study	Category	AI Domain	Species	Clinical Problem	Dataset	AI Method
Hubbard-Perez et al. (2024)	Peer-reviewed primary veterinary digital pathology/deep learning study.	Digital pathology / oncology	Dogs	Classification of hyperplastic lymph node and common canine lymphoma subtypes.	1,530 high-resolution microscopic images from canine lymph nodes/lymphoma subtypes.	Convolutional neural networks/deep learning

2 Supplementary Table S1.B Evidence Extraction

Table 2: Supplementary Table S1..B Evidence Extraction

Study	Validation	Metrics	Key Findings	Reported Limitations
McDonald et al. (2024)	Train/test split on canine recordings; cardiologist murmur grade and echocardiographic disease staging used as reference.	Murmur detection sensitivity 87.9%, specificity 81.7%; exact grade match 57.0%; B1/B2 MMVD AUC 0.861.	The model detected murmurs and helped differentiate stage B1 from B2 MMVD, supporting low-cost screening in primary care.	Referral-center population; exact grading remained imperfect; external/general-practice validation needed.
Jin et al. (2025)	Evaluation on generated records and independent real-world consultation records.	Generated dataset Top-3/5/10 disease prediction 91.4/93.4/95.9%; symptom extraction 79.9%; real-world Top-3/5/10 38.6/43.6/51.5%.	AVA improved over baselines and highlighted a major real-world performance drop, demonstrating both promise and translational risk.	Real-world records reduced performance substantially; dependence on symptom database quality; limited clinical deployment validation.
Arockiaraj et al. (2025)	Prototype/system-level evaluation; clinical validation unclear.	Not consistently reported in extracted text.	Proposes a scalable livestock monitoring system combining vital-sign sensors, GPS, and AI alerts.	Limited empirical validation, unclear dataset size, and unclear independent testing
Jana et al. (2025)	Model evaluation on accelerometer-derived behavior/activity data; validation design to verify from full text.	Performance metrics reported in paper; exact values to verify.	Shows how sensor data and explainable ML can support cattle activity/health monitoring.	Generalizability and farm-scale deployment require further validation.
Jarkoff et al. (2023)	Comparison with reference measurements; large-scale predictive performance evaluation.	Accuracy/performance metrics reported; exact values to verify.	Assesses the reliability of wearable-derived HR/BR measurements at scale.	Potential device, population, and reference-measurement constraints.
Hwang et al. (2022)	Internal model validation; exact split/cross-validation to verify.	Accuracy/sensitivity/specificity or related metrics reported; exact values to verify.	Demonstrates feasibility of multispectral deep learning for canine dermatology classification.	Likely limited dataset size, single setting, and need for external validation.
Panigrahy et al. (2025)	Model comparison with validation; exact approach to verify.	Accuracy/performance metrics reported; exact values to verify.	Compares classical ML and DL models for disease/risk prediction.	May rely on public/benchmark datasets and have limited clinical validation.

Study	Validation	Metrics	Key Findings	Reported Limitations
Ndiaye et al. (2025)	Comparison of AI interpretations with veterinary radiologist interpretations/reference classifications.	Accuracy, sensitivity/specificity, and/or finding-level performance; exact values to verify.	Provides real-world comparative evidence on commercial AI radiology performance.	Potential finding-level ambiguity, limited generalizability, and dependence on reference standard.
Görisch (2020)	Direct comparison with Holter-ECG reference.	Agreement/accuracy metrics for HR and HRV; exact values to verify.	Evaluates accuracy of wearable measurements against a clinical reference standard.	Device-specific performance, sample constraints, and activity/context effects.
Zhao et al. (2025)	Internal validation/test data; exact validation approach to verify.	Classification performance metrics reported; exact values to verify.	Demonstrates potential for DL classification of canine middle ear disease from CT images.	Likely limited external validation and dataset constraints.
Bhogapurapu et al. (2025)	Internal validation; exact method unclear.	Reported accuracy/performance; exact values to verify.	Demonstrates DL-based dog skin disease detection.	Likely limited dataset size and external validation.
Chinnam et al. (2025)	Internal validation; exact details to verify.	Accuracy/performance metrics reported; exact values to verify.	Provides additional evidence for image-based canine dermatology AI.	Possible dataset overlap, limited external validation, and prototype-level reporting.
Turner, Patel (2023)	Prototype demonstration; clinical validation unclear.	Not clearly reported.	Illustrates low-cost IoT monitoring for livestock health/productivity.	Limited AI transparency and validation.
Kim et al. (2023)	Model training/validation with EHR data; exact external validation to verify.	AUC/accuracy/sensitivity/specificity or related metrics reported.	Demonstrates use of EHR data for canine cardiac disease risk prediction.	EHR data quality, retrospective design, and generalizability constraints.
Biswas (2019)	Case-study demonstration.	Not consistently reported.	Shows a potential media-analysis approach for pet health diagnosis.	Limited peer-reviewed clinical validation and unclear dataset details.
Buric, Ivasic-Kos (2024)	Internal model evaluation; external validation to verify.	Segmentation/classification metrics reported; exact values to verify.	Demonstrates next-generation CV methods for canine ophthalmology.	Need for broader validation and clinical integration assessment.
Koo et al. (2021)	Model training/validation on retrospective clinical data; exact split/CV to verify.	Predictive accuracy/survival performance metrics; exact values to verify.	Combining multiple data types improved ML prediction of chemotherapy outcomes compared with conventional stratification.	Retrospective/single-cohort constraints and need for prospective validation.
Debes et al. (2023)	Train/evaluation on insurance-claims cohort.	Disease-category predictive performance; high accuracy reported across many categories.	Demonstrates the potential of large-scale insurance data for canine health prediction across 45 disease categories.	Claims data may be biased by insurance coverage, coding, breed reporting, and care-seeking behavior
Sholehrasa et al. (2025)	Model validation on real-world data; exact validation approach to verify.	Classification performance metrics reported; exact values to verify.	Shows potential for real-world regulatory data and XAI to support veterinary safety profiling.	Passive adverse event reporting biases, missing data, and causal inference limitations.

Study	Validation	Metrics	Key Findings	Reported Limitations
Dourson et al. (2023)	Model validation on ECG data; exact split/external validation to verify.	Classification metrics reported; exact values to verify.	Demonstrates automated ECG classification potential for veterinary cardiology decision support.	Need for broader clinical validation, multi-center testing, and arrhythmia-subtype performance assessment.
Wang (2024)	Mixed quantitative/qualitative user validation.	Owner satisfaction, monitoring accuracy, anxiety reduction; exact measures unclear.	Suggests potential benefits of pet wearables for monitoring and owner reassurance.	Commercial/product focus, unclear AI methods, and limited clinical validation.
Hubbard-Perez et al. (2024)	Training and validation dataset; exact split/test design to verify.	Classification performance metrics reported; exact values to verify.	Shows feasibility of DL for canine lymphoma subtype classification in digital pathology.	Preliminary study, image-level dataset constraints, and need for external/prospective validation.
